# Correlation between *Mycoplasma pneumoniae* drug resistance and clinical characteristics in bronchoalveolar lavage fluid of children with refractory *Mycoplasma pneumoniae* pneumonia

**DOI:** 10.1186/s13052-022-01376-6

**Published:** 2022-11-26

**Authors:** Xiao-Wen Zhan, Li-Ping Deng, Zhi-Yuan Wang, Ju Zhang, Meng-Zhu Wang, Shu-Jun Li

**Affiliations:** Department of Pediatrics, The First Affiliated Hospital of Xinxiang Medical University, No. 88 of Jiankang RoadHenan Province, Weihui, 453100 China

**Keywords:** Refractory Mycoplasma pneumoniae pneumonia, Drug-resistance gene mutation, Fibreoptic bronchoscopy, Alveolar lavage, Children

## Abstract

**Background:**

To investigate the resistance-gene mutation of *Mycoplasma pneumoniae* (MP) in the bronchoalveolar lavage fluid of children with Mycoplasma pneumoniae pneumonia (MPP) and the clinical characteristics of refractory Mycoplasma pneumoniae pneumonia (RMPP) correlation.

**Methods:**

Forty-eight children with MPP were selected and placed in RMPP and non-RMPP groups based on their clinical status – whether they had worsening clinical symptoms, persistent fever and a worsening lung image. They were also separated into drug-resistance gene mutation and non-mutated groups using nucleic acid detection. The participants’ data were collected on high-sensitivity C-reactive protein and MP-DNA loads, fever time, hospitalisation time, macrolide antibiotic application time and fever regression time after application. The differences in imaging manifestations were determined by using multivariate logistic regression to analyse the clinical characteristics of RMPP. Additionally, the correlation between drug-resistance gene mutations and the clinical characteristics of RMPP was summarised.

**Results:**

Among the 48 MPP children, 31 (64.6%) had *A2063G* and/or *A2064G* gene mutation, 31 (64.6%) had RMPP and 23 (74.2%) had drug-resistance gene mutation. The children in the drug-resistance gene mutation group had higher high-sensitivity C-reactive protein and MP-DNA loads, longer fever time, hospitalisation time, macrolide antibiotic application time, fever regression time after application and extrapulmonary complications. There were more symptoms and more severe changes under bronchoscopy. The difference was statistically significant (*P* < 0.05). Logistic multivariate regression analysis showed that the mutation of drug-resistance genes had no significant correlation with RMPP.

**Conclusion:**

The mutation rate of drug-resistance genes in children with MPP is high, the inflammatory index and MP-DNA load are high, the course of the disease is long, and the changes under bronchoscopy are severe. The occurrence of RMPP is not only determined by drug-resistance genes but may also be the result of a combination of factors.

## Introduction

*Mycoplasma pneumoniae* (MP) is classified as a pathogenic microorganism that is between bacteria and viruses. It is one of the most common community-acquired pneumonia (CAP) pathogens in children and can cause various clinical symptoms and even endanger their lives [1]. Mycoplasma pneumoniae pneumonia (MPP) accounts for about 10%–30% of CAP in children. In recent years, its incidence has been gradually rising [2, 3]. Currently, macrolides are the first choice of therapeutic drugs for treating MPP in children and have always had good efficacy. However, with the widespread use of macrolides in clinical practice, resistance to them is becoming more serious, and refractory Mycoplasma pneumoniae pneumonia (RMPP) cases are increasing [4]. The mechanism of MP resistance is still unclear; the reasons may be as follows [5, 6]: ① Gene site mutation: mainly related to the point mutation of the structural domain V of the *23S rRNA* gene, resulting in reduced binding to macrolides. The most common point mutations are *A2063G* and *A2064G*. ② Target site methylation: erm-encoded methylase can catalyse the methylation or demethylation of adenine at position 2058 of erythromycin and bacterial binding sites, affecting the binding of the two sites and leading to the development of drug resistance. ③ Active efflux system: the carried efflux pump may belong to the ABC transporter. (It is not clear whether it directly originates from *Enterococcus faecalis* or other transfer pathways. ④ Drug inactivation: bacteria have been shown to disrupt macrolide antibiotic activity by producing a passivating enzyme against them. RMPP is closely related to drug-resistance gene mutation and may also be related to some other factors. Thus, in this paper, we focus on the correlation between mutations in MP drug-resistance genes *A2063G* and *A2064G* in alveolar lavage fluid of children with MPP and RMPP and explore the related factors of RMPP. The results of this study will provide support for early intervention.

## Materials and methods

### Research subjects

This was a retrospective study. A total of 48 children with MPP were hospitalised in the pediatric department of the First Affiliated Hospital of Xinxiang Medical College and underwent alveolar lavage in the pediatric intensive care unit during the acute phase (within two weeks of the disease) from January 2019 to June 2021. They were selected for this study, and their clinical data were collected. The ethics committee of our hospital approved this study (No. 2018128), and informed consent was obtained from the families.

#### Inclusion criteria and exclusion criteria

Inclusion criteria: The diagnosis of MPP corresponded to the diagnostic criteria of the Expert Consensus on the Diagnosis and Treatment of MPP in Children (2015 edition) [7]. To obtain each child’s clinical picture, fibreoptic bronchoscopy was performed per the Chinese guidelines for paediatric bendable bronchoscopy (2018 edition) [8]. The participants’ families agreed to have the test performed and signed the consent form. Exclusion criteria: (1) incomplete clinical data; (2) chronic lung diseases (bronchopulmonary dysplasia, congenital malformation of the lung), bronchial foreign bodies, congenital heart disease, autoimmune deficiency and other basic diseases; (3) combinations with other pathological infections.

#### Grouping method

(1) RMPP group: those with worsening clinical signs, persistent fever, and worsening lung imaging after seven days or more of regular treatment with macrolide antibiotics; (2) non-RMPP group: those with improvement within seven days of regular treatment with macrolide antibiotics; (3) drug-resistant gene mutation group: those with *A2063G* and/or *A2064G* point mutations in the structural domain V of *23SrRNA*; (4) no mutation group: no *A2063G* and/or *A2064G* point mutation occurred.

#### General information

Clinical data were collected from 48 children with MP, including age, sex, white blood cell count, ultrasensitive C-reactive protein, procalcitonin, lactate dehydrogenase, throat swab and alveolar lavage fluid MP-DNA load, duration of fever, duration of cough, the number of days hospitalised, duration of macrolide antibiotic application and time to fever resolution after drug administration, fibreoptic bronchoscopic changes, the severity of pulmonary imaging, the extent of pulmonary infiltrates (multiple lobe involvement or ≥ 2/3 of the lung) [7] and extrapulmonary complications. The macrolide antibiotics used in each group were Azithromycin. After a diagnosis of RMPP, human immunoglobulin and hormones were used as adjuvant therapy. The methylprednisolone was used when the patient was febrile (2 mg/kg, once a day with withdrawal after three days).

### Experimental method

#### Alveolar lavage fluid collection

The bronchoscopy was performed in the acute stage (within two weeks). The same operator examined all patients. Bronchoalveolar lavage was performed with 37℃ saline (1 mL/kg per lavage) using fibreoptic bronchoscopy (model: CV-290) produced by Olympus, Japan. The specimens were obtained by negative pressure suction into a sterile sputum collector, then stored in a refrigerator at − 80℃ after timely processing for testing and sent for mycoplasma DNA detection (FQ-PCR method). The bronchoscopy can determine whether the patient has the following symptoms——Plastic bronchitis: a disease in which endogenous foreign bodies appear in the trachea, causing partial or total tracheal blockage and pulmonary ventilation dysfunction. Phlegm thrombus: bronchial secretions increase and stay in the trachea for a long time, resulting in airway blockage. Hyperemia: engorgement of submucosal capillaries with an abundant amount of blood. Oedema: a condition characterised by mucosal swelling and a smooth surface. Both are mainly inflammatory changes. Mucosal fold: a wrinkle on the surface of a mucous membrane. Mucosal erosion: localised superficial defect of mucosal epithelium leading to decay.

#### Nucleic acid extraction or purification and fluorescent quantitative polymerase chain reaction (PCR) techniques

The nucleic acid extraction or purification kit (Jiangsu Mole Bioscience Co., Ltd., China) and MP nucleic acid and drug-resistance mutation site detection kit (fluorescent PCR method) (Jiangsu Mole Bioscience Co., Ltd., China) were used, and the reagent instructions were strictly followed. Fluorescent quantitative PCR steps: (1) For sample processing, please refer to the instructions accompanying the nucleic acid extraction kit. When adding samples, add the internal standard simultaneously (5 μL/person). Mm, strong positive quality control, and mm, weak positive quality control, were processed in parallel with the samples. Normal saline was used as the negative control. (2) To prepare the amplification reagent, take the buffer, primer–probe, and water out of the kit; melt them on ice or at 4℃; take out the enzyme; shake it slightly; mix well; and centrifuge briefly at a low speed. Take out a sterile nuclease-free centrifuge tube and mark it. The reaction system is configured as follows: buffer 6.0 μL, primer–probe 2.0 μL, enzyme 0.5 us, water 11.5 μL. The total number of PCR reactions shall be the sum of the total number of samples: two positive QCs, one QC, one negative extraction control, and one PCR reaction negative control. (3) Add the prepared sample 5.0 into the PCR reaction tube containing PCR reaction solution μL (including the sample, strong and weak positive QCs, QC W and extraction negative control). Add the 5.0 at the same time as μL sterile water is used as the negative control of the PCR reaction. (4) To obtain the PCR reaction, put the reaction tube into the fluorescent PCR detector and set the cycle parameters as follows (Table 1).

**Table 1:** PCR cycle parameters

Number of cycles	Temperature (℃)	Reaction time(min:sec)
1	50	2:00
1	95	2:00
40	91	00:15
40	64	1:00

### Statistical analysis

SPSS 25.0 software was used for analysis. Quantitative data satisfying normal distribution and equal variance were expressed as mean ± standard deviation (x ± s). The independent samples t-test was used to compare the two groups; median (quartiles) was used for non-normal distribution; the non-parametric rank-sum test was used to compare groups; qualitative data were expressed as percentages (%); and the chi-square test was used for comparison between groups. Variables that were statistically significant in the univariate analysis and those that were professionally considered to affect the outcome were included in a multifactorial logistic regression model to explore independent influences on the outcome. Differences were considered statistically significant at *P* < 0.05.

## Results

### Analysis of clinical characteristics

#### RMPP group vs non-RMPP group

**Fig. 1:**
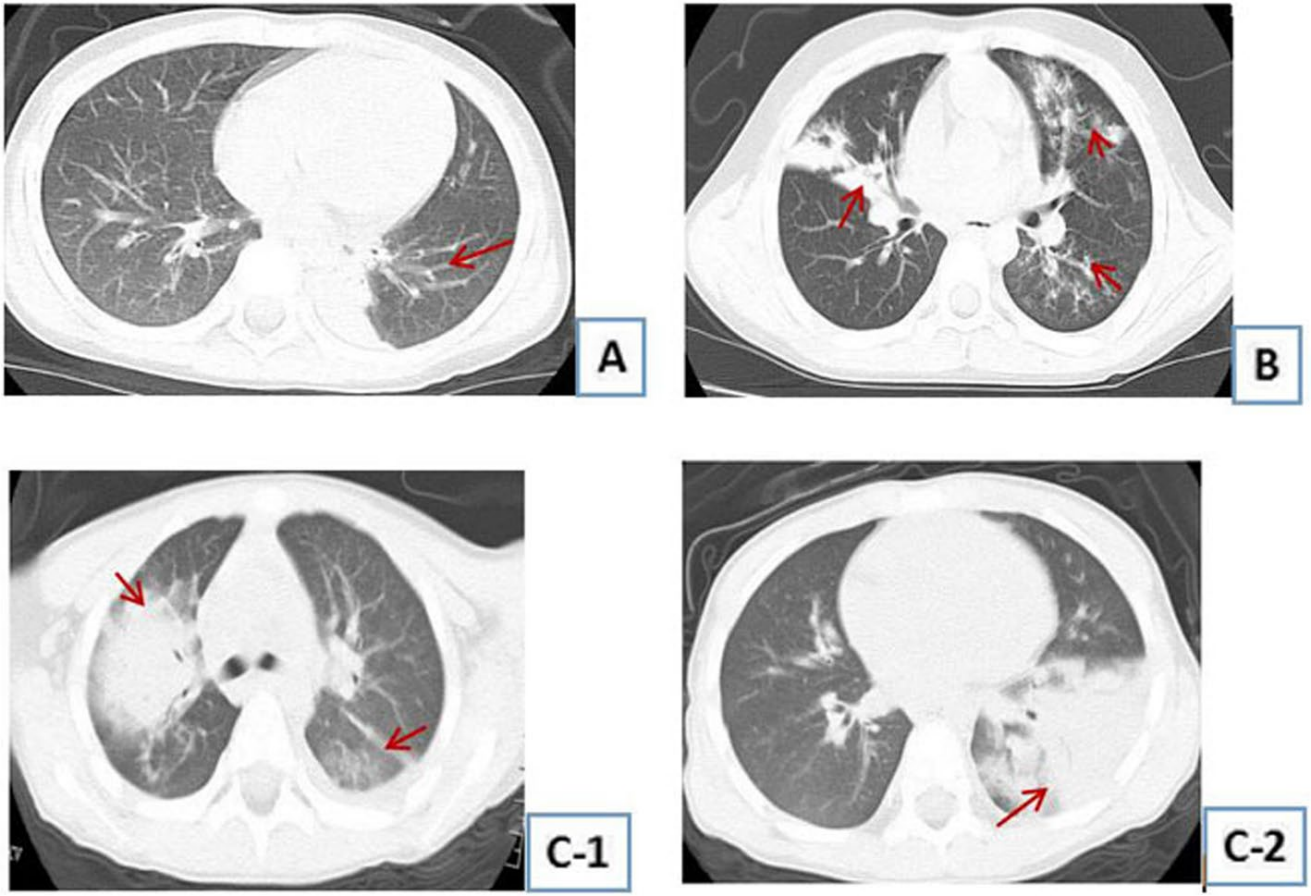
CT imaging changes of the lung. **A** Unilobar pulmonary consolidation high-density shadow (female, 4 years old). **B** Multilobar pulmonary consolidation high-density shadow (female, 4 years old). C-1、C-2.Pulmonary consolidation high-density shadow at different levels. (male, 4 years old)

As seen in Tables 2 and 3, the ultrasensitive C-reactive protein was higher in the RMPP group; the duration of fever, cough, hospital stay, macrolide application, and time to fever resolution after drug administration were longer. There were more extrapulmonary complications, and pulmonary imaging changes were more evident (see Fig. 1), with statistically significant differences (*P* < 0.05).

**Table 2:** Clinical characteristics of RMMP and non-RMPP

Clinical Features	Non-RMMP group (*n* = 17)	RMMP group (*n* = 31)	*P* value
Age (years)	4.79 ± 3.2	4.79 ± 3.47	0.983
MP-DNA × 10^5^ (copies/mL)	7. 83 ± 1.78	35. 81 ± 12.48	0.005
White blood cell count (× 10/L^9^)	7.28 ± 5.93	7.64 ± 3.17	0.192
Procalcitonin (ng/mL)	1.75 ± 3.29	1.52 ± 1.89	0.628
Ultrasensitive C-reactive protein (mg/mL)	18.06 ± 31.09	45.83 ± 64.69	0.001
Lactate dehydrogenase (U/L)	495.29 ± 199.19	795.97 ± 721.94	0.219
Duration of cough (days)	13 ± 8.36	18.03 ± 7.49	0.007
Fever duration (days)	7.82 ± 5.05	13.39 ± 7.65	0.009
Length of hospitalization (days)	9.12 ± 3.35	17.16 ± 7.08	0.000
Duration of macrolide application (days)	4.71 ± 2.47	11.61 ± 3.58	0.000
Time to fever remission after macrolide application (days)	3.69 ± 3.32	6.48 ± 4.01	0.021

*P* > 0.05 indicates no statistically significant difference between the two groups, and *P* < 0.05 indicates a statistically significant difference between the two groups

**Table 3:** Chi-square test for categorical variables between mutated and non-mutated groups, RMPP and non-RMPP groups

Indicators	Category	Mutated group	Non- mutated group	*P* value	Non-RMMP group	RMMP Group	*P* valuee
Gender	Male	22	10	0.393	11	21	0.831
	Female	9	7		6	10	
Imaging changes	Unilobar	12	11	0.085	13	10	0.003
	Multilobar	19	6		4	21	
Extra-pulmonary complications	None	14	13	0.037	14	13	0.007
	There are	17	4		3	18	

*P* > 0.05 indicates no statistically significant difference between the two groups, and *P* < 0.05 indicates a statistically significant difference between the two groups

#### Mutated and non-mutated groups

Compared with the non-mutated group, children in the drug-resistant mutated group were older and had a higher MP-DNA load in alveolar lavage fluid, a higher ultrasensitive C-reactive protein level, a longer duration of fever and hospital stay, a longer duration of macrolide antibiotic application and time to fever remission after application and more extrapulmonary complications with statistically significant differences of *P* < 0.05.

### Changes under fibreoptic bronchoscopy

#### RMPP group vs non-RMPP group

**Fig. 2:**
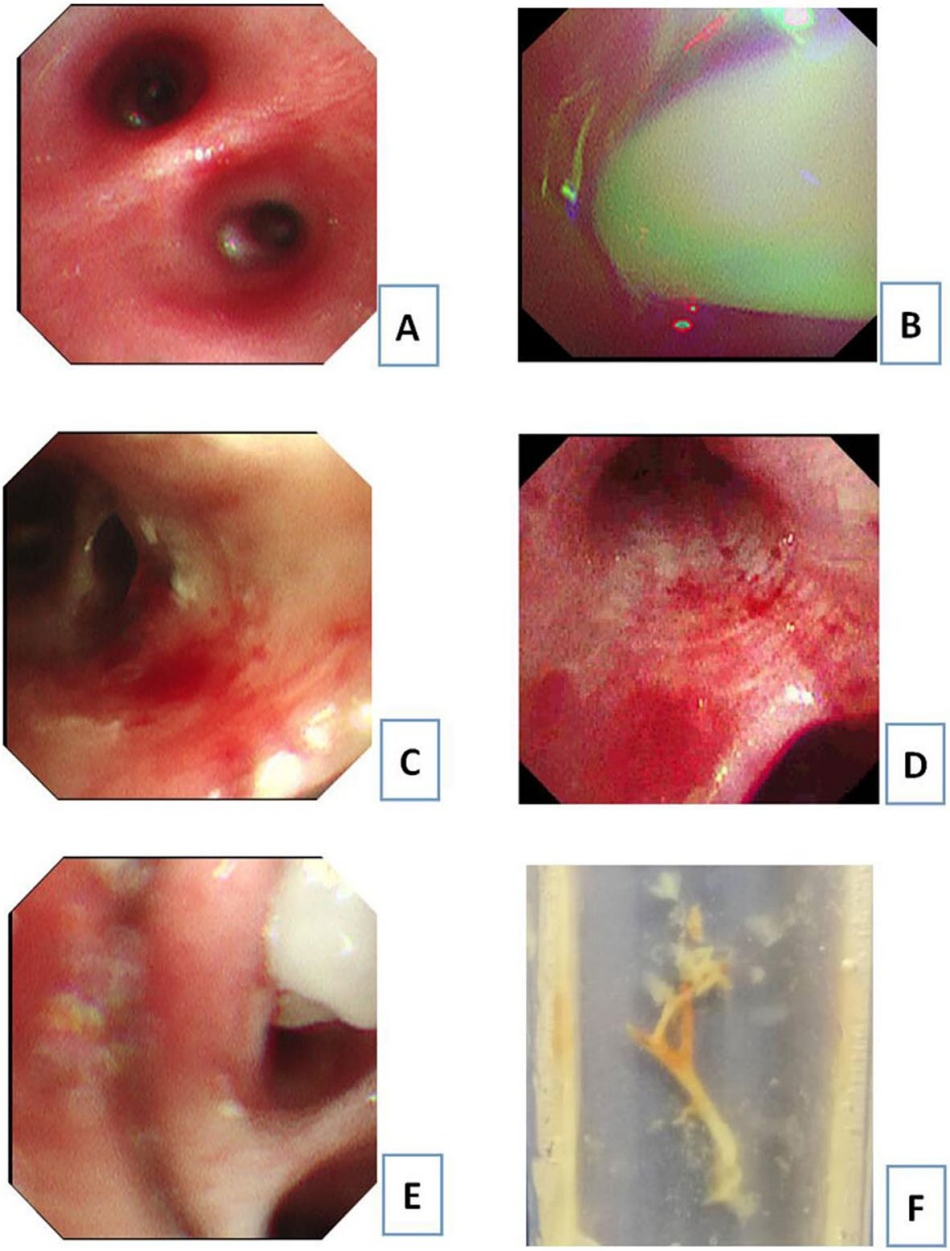
Changes under bronchoscopy. **A** Mucosal congestion and edema. **B** Sputum emboli. **C** Mucosal erosion. **D** Mucosal folds. **E** Molded sputum emboli. **F** plastic bronchi

In this study, the changes revealed by bronchoscopy were mainly mucosal congestion and oedema, sputum emboli, plastic bronchi, mucosal folds and mucosal erosion and necrosis. All children had mucosal congestion and oedema (see Fig. 2). All children with RMPP showed all five of these manifestations, with significantly more sputum emboli visible than in the non-RMPP group (*P* < 0.05), which was statistically significant (see Tables 4 and 5). There were two cases of plastic bronchi and three cases of mucosal folds in the RMPP group, neither of which was seen in the non-RMPP group.

**Table 4:** Clinical characteristics of the mutated and non-mutated groups

Clinical Features	Non-mutated group (*n *= 17)	Gene mutated group (*n* = 31)	*P* vaue
Age (years)	3.77 ± 3.90	5.35 ± 2.92	0.035
MP-DNA × 10^5^(copies/mL)	6. 59 ± 2.41	35. 88 ± 12.47	< 0.001
White blood cell count (× 10/L^9^)	6.91 ± 2.89	7.85 ± 4.91	0.635
Procalcitonin (ng/mL)	1.23 ± 1.81	1.81 ± 2.74	0.140
Ultrasensitive C-reactive protein (mg/mL)	23.85 ± 42.26	42.66 ± 62.45	0.030
Lactate dehydrogenase (U/L)	686.06 ± 635.33	691.36 ± 600.13	0.796
Duration of cough (days)	15.06 ± 7.69	16.90 ± 8.35	0.545
Fever duration (days)	9.12 ± 7.83	12.68 ± 6.80	0.024
Length of hospitalization (days)	12.00 ± 7.21	15.58 ± 6.88	0.027
Duration of macrolide application (days)	7.47 ± 3.47	10.10 ± 4.96	0.034
Time to fever remission after macrolide application (days)	3.88 ± 3.87	6.47 ± 3.79	0.023

*P* > 0.05 indicates no statistically significant difference between the two groups, and *P* < 0.05 indicates a statistically significant difference between the two groups

**Table 5:** Comparison of microscopic bronchial mucosa in the RMPP and non-RMPP groups

	Congestion and edema	Sputum emboli	Plastic Bronchi	Mucosal folds	Mucosal erosion
Non-RMPP group	17(100%)	3 (17.6%)	0(0)	0(0)	1(5.9%)
RMPP group	31(100%)	15 (48.4%)	2(6.5%)	3 (9.7%)	4 (12.9%)
Card Parties		8.000			1.800
*P* value		0.005			0.180

*P* > 0.05 indicates no statistically significant difference between the two groups, and *P* < 0.05 indicates a statistically significant difference between the two groups

#### Gene-mutated group vs non-mutated group

Bronchoscopic sputum emboli manifestations were still significantly more frequent among children in the gene mutation group than in the non-mutated group (*P* < 0.05), which was statistically significant (see Table 6). The few manifestations of plastic bronchi, mucosal folds and erosion found in the mutated group were not found in the non-mutated group.

**Table 6:** Comparison of microscopic bronchial mucosa in the mutated and non-mutated groups

	Congestion and edema	Sputum emboli	Plastic bronchus	Mucosal folds	Mucosal erosion
Non-mutated group	17(100%)	4 (23.5%)	0(0)	0(0)	0(0)
Gene mutated group	31(100%)	14 (45.2%)	2(6.5%)	3 (9.7%)	5 (16.1%)
Chi-square		5.556			
*P*		0.018			

*P* > 0.05 indicates no statistically significant difference between the two groups, and *P* < 0.05 indicates a statistically significant difference between the two groups

### Relationship between drug-resistant gene mutations and RMPP

*A2063G* and/or *A2064G* mutations occurred in 31 of 48 children with MPP, accounting for 64.6%; 23 of 31 children with RMPP had *A2063G* and/or *A2064G* drug-resistant mutations, accounting for 74.2%; 8 had no mutations, accounting for 25.8%; 8 of 17 children with non-RMPP had drug-resistant mutations, accounting for 47.1%; and nine had no drug-resistant mutations, accounting for 52.9%. Statistical analysis of the chi-square test (*P* = 0.065 (*P* > 0.05) did not show any statistical significance for drug-resistant gene mutation and RMPP (see Tables 3 and 7).

**Table 7:** Multi-factor logistic regression analysis of clinical characteristics of RMPP

Related factors	B	S.E	Wald	*P* value	OR	95% CI	
						Lower limit	Upper limit
Gene mutation	-1.174	0.636	3.406	0.065	0.309	0.089	1.076
Duration of macrolide use	1.378	0.429	10.329	0.001	3.968	1.712	9.198

### Multi-factor logistic regression analysis of clinical characteristics of RMPP

Logistic regression analysis of statistically significant indicators in both RMPP and non-RMPP groups of cases showed that the drug-resistance gene mutations were not significantly correlated with RMPP (see Table 7).

## Discussion

In this study, drug-resistance gene mutations occurred in 31 of the 48 children with MPP (64.6%). There may be geographical differences, with resistance rates varying from region to region [9].

It has been reported that children with severe MPP infection may experience encouraging results [10] when other effective antibiotic alternative treatments, such as quinolones and tetracyclines, are administered. These drugs, which can be a good choice for adults, should carefully be considered when prescribed for children. Drug-related adverse effects should be considered in conjunction with the child’s condition. In this study, 48 children with MPP were eventually treated effectively and recovered well, probably because of the following: ① MPP is a self-limiting disease; ② mutations in drug-resistant genetic loci are not the only possible resistance mechanisms occurring in RMPP, in which autoimmune reactions may be involved. A reliable treatment option, especially for severe MPP infection, is the administration of human immunoglobulin and hormones because they can inhibit excessive immune reactions; ③ the diagnostic and therapeutic value of fibreoptic bronchoscopy and alveolar lavage is high. The advantages are obvious: alveolar lavage prevents further exacerbation and is suitable for treating clinical symptoms and improving lung imaging [11].

Among the four kinds of mechanisms of MP resistance, the study of 23S rRNA gene mutation leading to the mutation of binding site is a hot spot. In 1995, French scholar Lucier et al. [12] found mutations at positions 2063 and 2064 in the V region of 23S rRNA in Macrolide resistant strains. Pereyre et al. [13] also detected 2058,2059 A to G point mutations in V region of 23S rRNA from MP resistant strains isolated in France. Japanese scholar Okazaki et al. [14] detected A to G point mutation at position 2063 in 15 MP-resistant induced strains isolated from clinic, point mutations at positions 2063 A to G and 2064 A to G or C were also detected in erythromycin-induced strains, and it was inferred that the resistance phenotype was closely related to the site of the point mutation. Chinese scholar Xin et al. [15] screened 50 strains of MP isolated from children and found 46 strains were resistant to Macrolide. The results of gene sequencing showed that 40 of these strains had mutations at 2063 A to G in V region of 23S rRNA, 5 had mutations at 2064 A to G, and 1 had mutations at 2063 A to C. Liu et al. [16] reported the presence of 44 resistant strains in 53 MP clinical isolates and all had mutations in the V region of 23S rRNA at positions 2063 A to G.

In this study, logistic multivariate regression analysis led to the conclusion that drug-resistant gene mutations were not correlated with RMPP, which is different from the results reported by Xu et al. [17]. There are more possible mechanisms for the occurrence of RMPP, including drug-resistant gene mutations, immune dysfunction, mixed infections, excessive MP load, a mucus plug, a hypercoagulable state and a community-acquired respiratory distress syndrome toxin. Drug-resistance gene mutation is not the only mechanism. The immune response generated by the organism may also play an important part [18]. The immune dysfunction of the organism after reactive infection with MP leads to a series of inflammatory reactions, resulting in increased lung injury and exacerbation of the disease, suggesting that the immune response of the organism after MP infection may be a factor that cannot be ignored in the development of RMPP.

In this study, we found that the children in the drug-resistant gene mutation group were predominantly five years and older, had a longer duration of fever, hospitalisation, macrolide antibiotic application, fever resolution after application and a higher alveolar lavage fluid MP-DNA load and ultrasensitive C-reactive protein. The findings of Zhan et al. [19] in 97 children with MPP (80 of whom were children with drug-resistant gene mutations) were consistent with the results of this study but differed from it in terms of age, lactate dehydrogenase, cough duration and pulmonary imaging changes. In this study, the age of children with drug-resistant gene mutation was older (around five years old), which was similar to the MP detection rate in the age group found by Li et al. [20]. The detection rate of the *A2063G* drug-resistant gene was the highest in the three-year-old ≤ age < seven-year-old group, which was 58.9%, considering that it may be related to the preferred age of children with MPP, with the peak incidence in children who are five to nine years old. There were differences in lactate dehydrogenase, cough duration, and pulmonary imaging changes, which may be because the bronchoalveolar lavage fluid obtained from the experimental samples in this study yielded a higher positive rate than the serum, sputum and pharyngeal wipe test samples. Chen et al. [21] found a correlation between mycoplasma DNA load and drug-resistance mutations in the lavage fluid of fibreoptic bronchoscopy lavage treatment in children with MPP in the mutation group, which is consistent with the results of this study, suggesting that elevated mycoplasma load may predict the development of drug resistance. In this study, it was observed that mucosal congestion and oedema were present under bronchoscopy in the drug-resistant gene mutation group, the occurrence of sputum emboli was significantly higher than that in the unmutated group, and plastic bronchitis, mucosal folds and erosions that occurred occasionally were found in the unmutated group. This finding was different from the conclusion that there was no difference between both in the previous study. In this study, we found that compared with the non-RMMP group, the RMPP group had longer fever, cough, hospitalisation and macrolide antibiotic duration; longer fever remission time after application; higher ultrasensitive C-reactive protein; more severe pulmonary imaging and bronchoscopic changes; and more extrapulmonary complications. Some findings indicate that a higher MP-DNA load is associated with a greater inflammatory response, more extrapulmonary complications and more severe disease [22], suggesting a possible association with the development of RMPP. It was observed that children with RMPP showed significantly more microscopic mucosal congestion and oedema, folds, erosions, ductal purulent secretions, sputum emboli, plastic sputum emboli, and ductal occlusion than children with common MPP.

This study has some limitations: it is a small sample study, and it involves only the common loci 2063 and 2064, while the existence of other loci mutations in drug-resistant strains and their resistance mechanisms remain to be explored in depth.

## Conclusion

In summary, children in the RMPP group and the drug-resistance gene mutations group have more severe clinical signs, higher inflammatory indexes, and a longer treatment course. In clinical practice, we can predict the occurrence of RMPP by monitoring the changes of the above indicators in children with MP at an early stage and intervening early to reduce the children’s pain and hospitalisation duration.

## Data Availability

The datesets used or analyzed during the current study are available from the corresponding author on reasonable request.

## Abbreviations

MP: *Mycoplasma pneumoniae*
MPP: Mycoplasma pneumoniae pneumonia
RMPP: Refractory Mycoplasma pneumoniae pneumonia
CAP: Community-acquired pneumonia
